# Proteomics approaches towards early detection and diagnosis of ovarian cancer

**DOI:** 10.1186/2051-1426-2-S1-O5

**Published:** 2014-02-24

**Authors:** Ali Tiss, John Timms, Usha Menon, Alex Gammerman, Rainer Cramer

**Affiliations:** 1 Dasman Institute, Biochemistry & Molecular Biology Unit, Dasman Institute, Kuwait City, Kuwait; 2 EGA Institute for Women’s Health, Proteomics Unit, University College London, London, UK; 3 Royal Holloway University of London, Computer Learning Research Centre, Royal Holloway, University of London, Egham, UK; 4 University of Reading, Department of Chemistry, University of Reading, Reading, UK

## 

Early stage detection of cancer is the key to provide a better outcome for therapeutic intervention. Proteomic technologies hold recently great promise in the search of new clinical biomarkers for the early detection and diagnosis of cancer or for the development of new vaccines.

In this perspective, we will present our recent work in improving early diagnosis of ovarian cancer (OC) by combining MS analysis of serum peptidome with data collected over a period of 7 years from the United Kingdom Collaborative Trial of Ovarian Cancer Screening. Using 295 patients with OC, 290 with benign neoplasm, and 2236 postmenopausal healthy controls, our results showed that OC could be accurately predicted up to 15 months before its clinical diagnosis, based a combination of two MS peaks with CA125 clinical test. An overall sensitivity of 94.8% (96.6% specificity) was obtained when comparing malignancies versus healthy postmenopausal controls. High classification accuracies were also obtained for early-stage cancers (93.5% sensitivity). MS discriminatory peaks were identified as connective tissue-activating peptide III (CTAPIII) and platelet factor 4 (PF4), platelet-derived chemokines, suggesting a link between platelet function and tumour development. Those markers might be promising for clinical use in cancer early detection and treatment.

